# Supplementary material for: Distinct pathways of homologous recombination controlled by the SWS1–SWSAP1–SPIDR complex
Source: Nat Commun. 2021 Jul 12;12:4255. doi: 10.1038/s41467-021-24205-6 (PMC8275761; doi:10.1038/s41467-021-24205-6)
Supplement: Supplementary file 1 — Supplementary Information [file 41467_2021_24205_MOESM1_ESM.pdf]

Distinct pathways of homologous recombination controlled by the SWS1-SWSAP1-SPIDR complex

Prakash et al.

Supplementary Information

Supplementary Figure 1

a

SWS1

```
human  MAVVLPAVVEELLSEMAAAVQESARIPDEYLLSLKFLFGSSATQALDLVD 50
mouse  MAVALPEVVEELLSEMAAAVRDSARIPDELLLSLEFVFGSSAIQALDLVD 50
       ***.** *************::******* ****:*:***** *******

                     β-strand   β-strand
human  RQSITLISSPSGRRLVYQVGSSSKTYTCLASCHYCSCPAFAFSVLRKSDS 100
mouse  RESVTLISSPSGRRVYQVLGSSGKTYTCLASCHYCSCPAFSFSVLRKSDS 100
       *:*:******************.*****************:*********

           α-helix
human  ILCKHLLAVYLSQVMRTCQQLSVSDKQLTDILLMEK------------KQ 138
mouse  LLCKHLLAIYLSQLLRNCQQLHVSDKQLTDLLMEDTRRIKGAAGTWTSKT 150
       :*******:****::*.**** ********:*: :.             *

human  EA 140
mouse  EA 152
       **
```

b

SWSAP1

```
                                        Short (annotated)
       Long                                     Walker A       FxxA
human  MAETLRRVLTRGGAAWSGEENMPAAGPPLLLLGTPGSGKTALLFAAALEAAG 52
mouse  MAEALRRVLNAGCAARPGEDG--AAGPPLLLLGAPRSAQTSLLFAAALEAAG 50
       ***:*****. * **  **:   **********:* *.:*:***********

human  EGQGPVLFLTRRPLQSMPRGTGTTLDPMRLQKIRFQYPPSTRELFRLLCS   102
mouse  EGRGSVLFVTRRPLQSLPLSTPTAREHWRLQKVRFQYPSSIQELLQLLAS   100
       **:*.***:*******:* .* *: :  ****:*****.* :**::**.*

                Walker B
human  AHEAPGPAPSLLLLDGLEEYLAEDPEPQEAAYLIALLLDTAAHFSHRLGP   152
mouse  AHEAPAPTPSLLLLDGLEEYLAEDPSAQEAAYLAALLLDTAAFFSHRLGA   150
       *****.*:*****************..****** ********.******.

human  GRDCGLMVALQTQEEAGSGDVLHLALLQRYFPAQCWLQPDAPGPGEHG-L   201
mouse  NGSCGLVVALETQKEA-EADAPHLPLLKRYFPAQCWLQPDALGLGQHCCL   199
       . .***:***:**:** ..*. **.**:************** * *:*  *

human  RACLEPGGLGPRTEWWVTFRSDGEMMIAPWPTQAGDPSSGK-GSSSGGQP   250
mouse  RASLELGKLSPRTEWSVSFLPCGEMKVTPWLAQASKLSPEKKDSSAGSQS   249
       **.** * *.***** *:* . *** ::** :**.. *. * .**:*.*.

human  ------------------------------
mouse  LTLGCDNLPGPGSPLDGILTSETGADSKT                        278
```

c

SPIDR

```
Human  MPRGSRARGSKRKRSWNTECPSFPGERPLQVRRAGLRTAGAAASLSEAWLRCGEGFQNTS 60
Mouse  MSGARRPGTSKRKRNWHIEHPSFREERSQQLRRGNFKTVEAADSLSKAWLKCGEGFQDTS 60
       *  . *   *****.*: * ***  **  *:**..::*. ** ***:***:******:**

Human  GNPSLTAEEKTITEKHLELCPRPKQETTTSKSTSGLTDITWSSSGSDLSDEDKTLSQLQR 120
Mouse  EILSLASEKTGITEKHLELSPKPKTETT-SKNASELPNIIWSSSESDFSDEDKTLPALQR 119
          **::*:. ********.*:** *** **.:* * :* **** **:*******  ***

Human  GNGHCSRIDRFCNRNILCPEDQSNEDELQFIDWEIDSDRAEASDCDEFEDDEGAVEISD 179
Mouse  DGRHGPRADRLGDRTISCPEDEDIEDELQVIDWEVNSDKEDPGGPSECEDDKGTLDISD 178
       .. *  * **: :*.* ****:. *****.****::**: : .. .* ***:*:::***

Human  CASCASNQSLTSDEKLSELPKPSSIEILEYSSDSEKEDDLENVLLIDSESPHKYHVQFAS 239
Mouse  CDSCAS---LTSDDRLCEPSEPISTEILEYSSDSEKEEDPEHSLFIDSESPHKYQADFKS 235
       * ****   ****::*.*  :* * ************:* *: *:*********:.:* *

Human  DARQIMERLIDPRTKSTETILHTPQKPTAKFPRTPENSA-KKKLLRGGLAERLNGLQNRE 298
Mouse  DARWCLVSQTDSEANSAEP-TLTPQKYTVKFPKTPEYSVTKKKLLRGGLAERLQELQNRK 294
       ***  :    * .::*:*    **** *.***:*** *. *************: ****:

Human  RSAISLWRHQCISYQKTLSGRKSGVLTVKILELHEECAMQVAMCEQLLGSPATSSSQSVA 358
Mouse  RSAISLWRHRCVSYQMTPLGRKSGVLTVKILELHEECSMQVAVCEQLAGPPITSPPGGLA 354
       *********:*:*** *  *****************:****:**** * * **   .:*

Human  PRPGAGLKVLFTKETAGYLRGRPQDTVRIFPPWQKLIIPSGSCPVILNTYFCEKVVAKED 418
Mouse  PRPGAYLKVLFTRETADHLMGHPQDIIYIFPPWQKLLIPNGSCSIILNTYFCQKAIAKET 414
       ***** ******:***.:* *:*** : ********:**.*** :*******:*.:***

Human  SEKTCEVYCPDIPLPRRSISLAQMFVIKGLTNNSPEIQVVCSGVATTGTAWTHGHKEAKQ 478
Mouse  VR--EDLYSPDISLSRRNITLAQTFRIKDITDNSSINQTTYDSLATPGTGWTHGHEKAEQ 472
        .   ::*.*** * **.*:*** * **.:*:**    *.. ..:** **.*****::*:*

Human  RIPTSTPLRDSLLDVVESQGAASWPGAGVRVVVQRVYSLPSRDSTRGQQGASSGHTDPAG 538
Mouse  HLIVAAPLRNSLLDIVESQRAGLWSGVRVQVVVQRVYSLLSRDGARSQQGHTVGHADASG 532
       :: .::***:****:**** *. * *. *:********* ***.:*.*** : **:* :*

Human  TRACLLVQDACGMFGEVHLEFTMSKARQLEGKSCSLVGMKVLQKVTRGRTAGIFSLIDTL 598
Mouse  AWSCLLVQDACGMFGEVFLNSTLWKSRQLEGKSCSMSGVKVLQKATRGRTPGLFSLIDSL 592
       : :**************.*: *: *:********: *:*********.***** *:*****:*

Human  WPPAIPLKTPGRDQPCEEIKTHLPPPALCYILTAHPNLGQIDIIDEDPIYKLYQPPVTRC 658
Mouse  WPPVISLTEPSCGQPSGETKTYLPPPIFCYIFSAHPTLGQIDAIE-DHISKLYQPPVVRC 651
       ***.* *. *. .**. * **:****  :***::***.***** *: * * *******.**

Human  LRDILQMNDLGTRCSFYATVIYQKPQLKSLLLLEQREIWLLVTDVTLQTKEERDPRLPKT 718
Mouse  LKEILQTNECSTRCSFYARVIYQKPQLKNL--LAQKEIWLLVTDITLQTQDERDHSLPKT 709
       *::*** *: .******* *********.*  * *:********:****::***  ****

Human  LLVYVAPLCVLGSEVLEALAGAAPHSLFFKDALRDQGRIVCAERTVLLLQKPLLSVVSGA 778
Mouse  LPVYIAPSCVLGPEVVEELALLVSYNLLFRDAFKDNGQIVCIERTVILPQKPLLCVP--S 767
       * **:** **** **:* **  . :.*:*:**::*:*:*** ***:* ****:* *****.*   :

Human  SSCELPGPVMLDSLDSATPVNSICSVQGTVVGVDESTAFSWPVCDMCGNGRLEQRPEDRG 838
Mouse  ASCDLPSPVTLDELSALTPVNSICSVQGTVVDVDESTAFSWPVCDRCGNGRLEQKPEDGG 827
       :**:**.** **.*.: **************.************* ********:*** *

Human  AFSCGDCSRVVTSPVLKRHLQVFLDCRSRPQCRVKVKLLQRSISSLLRFAAGEDGSYEVK 898
Mouse  TFSCGDCSQLVLSPLQERHLHVFLDCPTRPESTVKVKLLESSISLLLMSAASEDGSYEVE 887
       :*******::* **: :***:***** :**:. ******: *** **  **.*******:

Human  SVLGKEVGLLNCFVQSVTAHPTSCI-GLEEIELLSAGGASAEH---      940
Mouse  SVLGKEMGPLLCFVQSITTQQSSCVVTLEEIELLSTEGATAAQPPP      933
       ******:* * *****:*:: :**:  ********: **:* :
```

d

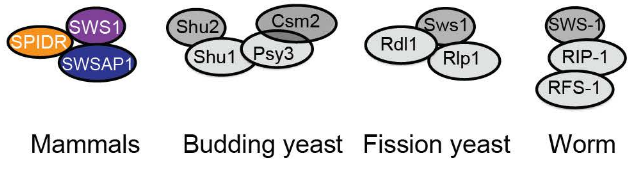

Supplementary Fig. 1: Mouse SWS1, SWSAP1, and SPIDR alignments with the human proteins.

a. Human and mouse SWS1 are 83% identical (96% similar). Each contains a conserved Zn-interacting domain with a CxC(x_{15})CxH structure, with the cysteine and histidine residues (red) flanked by two beta-strands and an alpha-helix (underlined) which contain hydrophobic residues characteristic of SWS1 proteins[8,9]. The secondary structure prediction was obtained using YASPIN software. Mouse SWS1 is predicted from the NCBI reference sequence to contain an extra 12 amino acids near the C terminus that were not present in a previous alignment[8]. RT-PCR performed on wild-type testes RNA confirms the presence of the coding sequence for these 12 amino acids.

b. Human and mouse SWSAP1 are 69% identical (90% similar). Both share a similar degree of identity with RAD51 (~18%) and are predicted to be RAD51 paralogs[10]. While both the human and mouse proteins contain a conserved Walker B motif (underlined), only the human protein contains a conserved Walker A motif (Gx_4GKT). The NCBI prediction for human SWSAP1, which was included in[10], has it initiating at the second ATG; however, a start at the first ATG in the transcript gives another 21 amino acids which match well with the mouse and other SWSAP1 proteins (e.g., chimp, macaque) and so we have included it in the alignment. Interestingly, a human *SWSAP1* variant allele disrupts the stop codon to continue translation for another 39 amino acids (WALVGDYLSVLQFPMA GILTSGTYADPKT; not shown), the last 13 of which are very similar to the mouse protein.

c. Human and mouse SPIDR are 65% identical (83% similar). The NCBI prediction for mouse SPIDR includes a different splice donor for exon 4 compared with the prediction of the human protein, extending the coding sequence for mouse exon 4 and leading to a gap in the alignment with the human protein (not shown). Using this splice donor for the human protein, however, restores the homologous sequence (shown in blue). Protein alignments were done using ClustalQ program.

d. Diversity of Shu complexes in different organisms. A SWS1 homolog is evident in each organism (dark gray), while the number and structure of RAD51-related proteins varies (light gray). See also[14]. The budding yeast complex is the only one for which a structure has been published[15].

Supplementary Figure 2

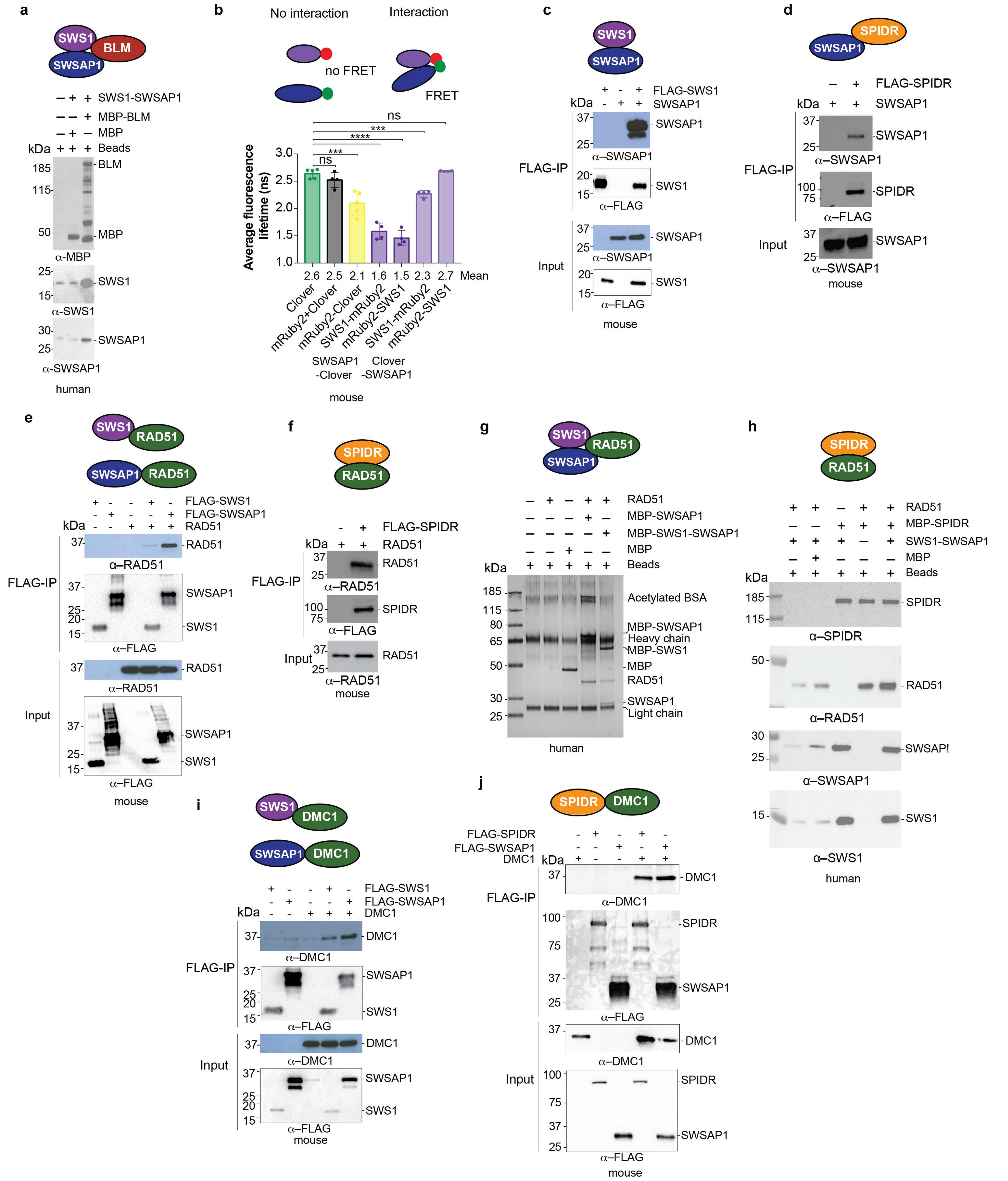

Supplementary Fig. 2: SWS1, SWSAP1, and SPIDR protein interactions.

a. SWS1-SWSAP1 interaction with BLM. MBP-BLM was pulled down with anti-MBP beads and interacting proteins were identified by western blotting with the indicated antibodies. All of the proteins are human. n=3, where n is the number of independent experiments.

b. FRET analysis shows that mouse SWS1 and SWSAP1 form a complex in RPE cells. An interaction with SWS1 is only observed when SWSAP1 is tagged at the C terminus. Clover and mRuby2-Clover fusion, n=5; mRuby2+Clover, SWS1-mRuby2 fusion+SWSAP1-Clover fusion, mRuby2-SWS1 fusion+SWSAP1-Clover fusion, SWS1-mRuby2 fusion+Clover-SWSAP1 fusion, mRuby2-SWS1 fusion+Clover-SWSAP1 fusion, n=4, where n is the number of independent experiments.

c-f. Interactions are observed for mouse SWSAP1 with SWS1 (c) and with SPIDR (d) and also between the three proteins with RAD51 (e,f) in co-immunoprecipitation experiments from HEK293 cells. FLAG-tagged proteins were pulled down with anti-FLAG beads and interacting proteins were identified by western blotting with the indicated antibodies. n=3, where n is the number of independent experiments.

g,h. RAD51 interactions are also observed with human SWS1-SWSAP1 (g) and SWS1-SWSAP1-SPIDR (h) using purified proteins. MBP- tagged proteins were pulled down with anti-MBP beads and interacting proteins were identified by western blotting with the indicated antibodies. n=3, where n is the number of independent experiments.

i,j. Interactions are observed for mouse SWS1-SWSAP1 (i) and SPIDR (j) with DMC1 in co-immunoprecipitation experiments from HEK293T cells. FLAG-tagged proteins were pulled down with anti-FLAG beads and interacting proteins were identified by western blotting with the indicated antibodies. n=3, where n is the number of independent experiments.Error bars in **b**, mean ± s.d. ***$P \leq 0.001$; ****$P \leq 0.0001$; unpaired t test, two-tailed.

All source data are provided in the Source Data file.

Supplementary Figure 3

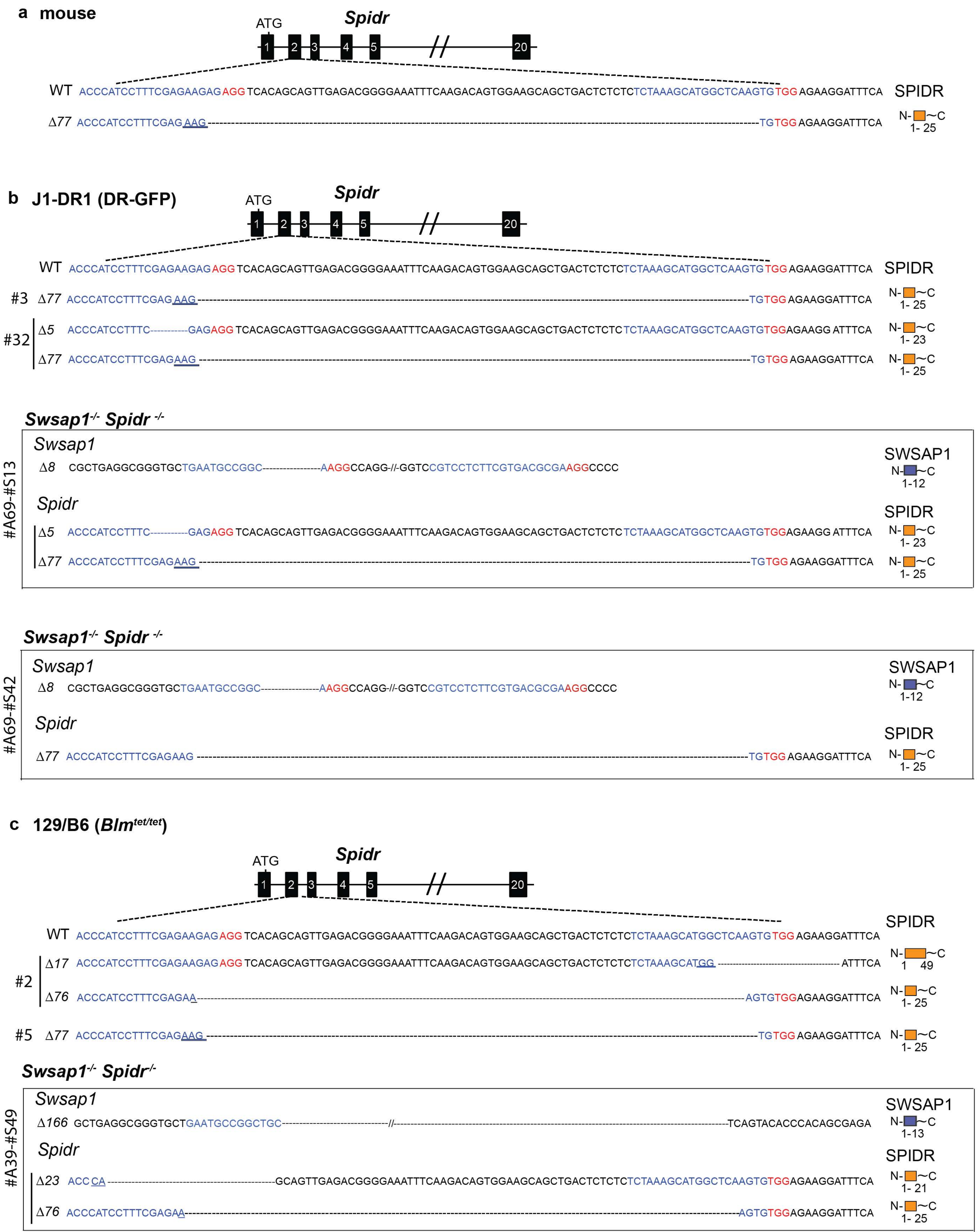

Supplementary Fig. 3: Mutated alleles analyzed for *Spidr* and *Swsap1 Spidr* in mouse ES cells and mice.

a,b,c. Frameshift mutations in *Spidr* in mice (a), *Spidr* and *Swsap1 Spidr* J1-DR1 (b), and *Spidr* and *Swsap1 Spidr* 129/B6 *Blm*$^{tet/tet}$ (c) ES cells. Genomic structure and sequence around the gRNA (blue) and PAM (red) sequences are shown. The number for each mutant clone is shown on the left, together with the detected mutated allele(s). The truncated protein product predicted from each mutated allele is diagrammed on the right. 129/B6 *Blm*$^{tet/tet}$ were used in c to be able to regulate BLM expression (-Dox, BLM is expressed; +Dox, BLM is not expressed).

Supplementary Figure 4

a

Spidr		
+/+	+/-	-/-
25 % (10)	50 % (20)	25% (10)

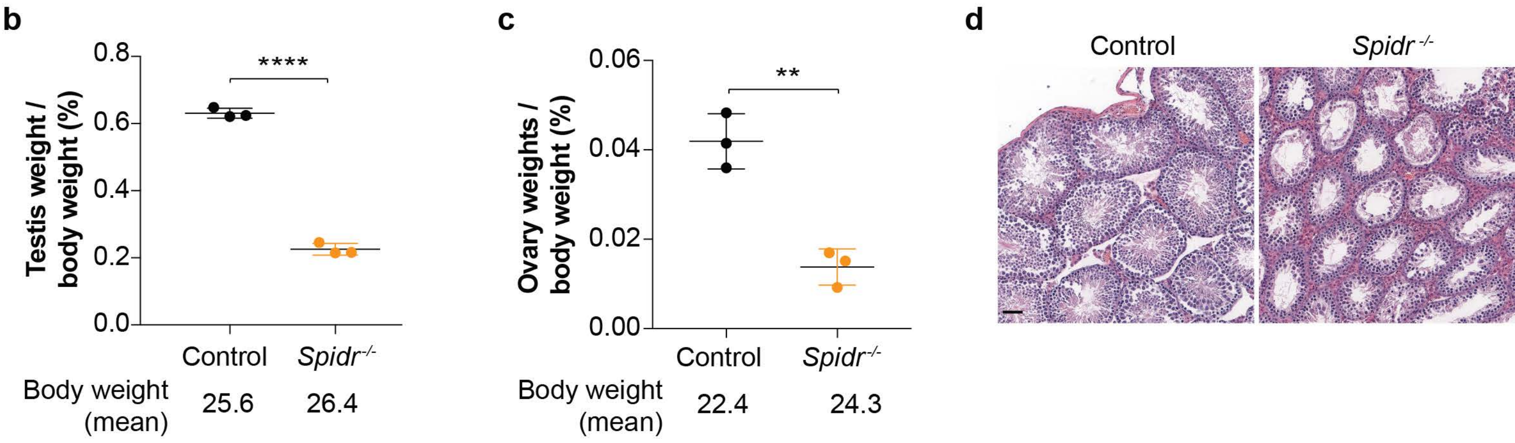

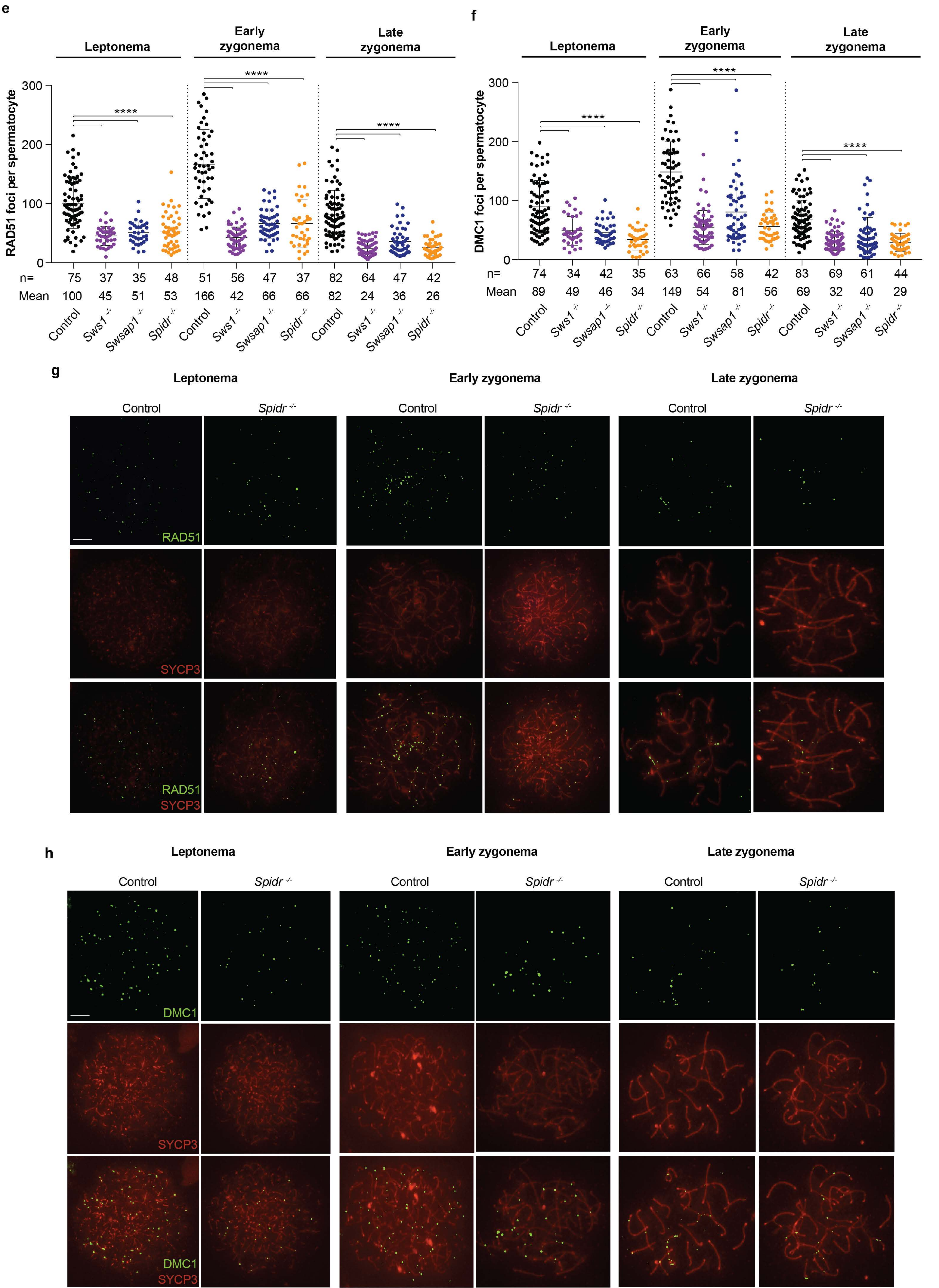

Supplementary Fig. 4: *Spidr* mutation leads to defect in meiotic HDR.

a. *Spidr*$^{-/-}$ homozygous mice are viable and born at normal Mendelian ratios.

b. Testes to body weight ratios are significantly reduced in *Spidr*$^{-/-}$ mice. n=3, where n is the number of mice.

c. Ovary to body weight ratios are significantly reduced in *Spidr*$^{-/-}$ mice. n=3, where n is the number of mice.

d. Testis section from a *Spidr* mutant showing seminiferous tubules with substantially reduced post-meiotic germ cells. Scale bar, 100 µm. n=3, where n is the number of mice.

e,f. RAD51 (e) and DMC1 (f) foci for data presented in **Fig. 1c,d (**n=2), combined with previously published data from controls, *Sws1,* and *Swsap1*[7]. The number of spermatocytes examined for each condition is indicated on the figure; spermatocytes are derived from 3 mice.

g,h. Representative chromosome spreads from adult mice from various stages of early prophase I from control and *Spidr* mutant spermatocytes to analyze RAD51 (g) and DMC1 (h) focus formation for data presented in **Fig. 1c,d**. Scale bar, 10 µm. The number of spermatocytes examined for each condition is indicated in panels e,f; spermatocytes are derived from 3 mice.

Error bars in **b,c,e,f**, mean ± s.d. $**P \leq 0.01$; $****P \leq 0.0001$; unpaired t test, two-tailed.

All source data are provided in the Source Data file.

Supplementary Figure 5

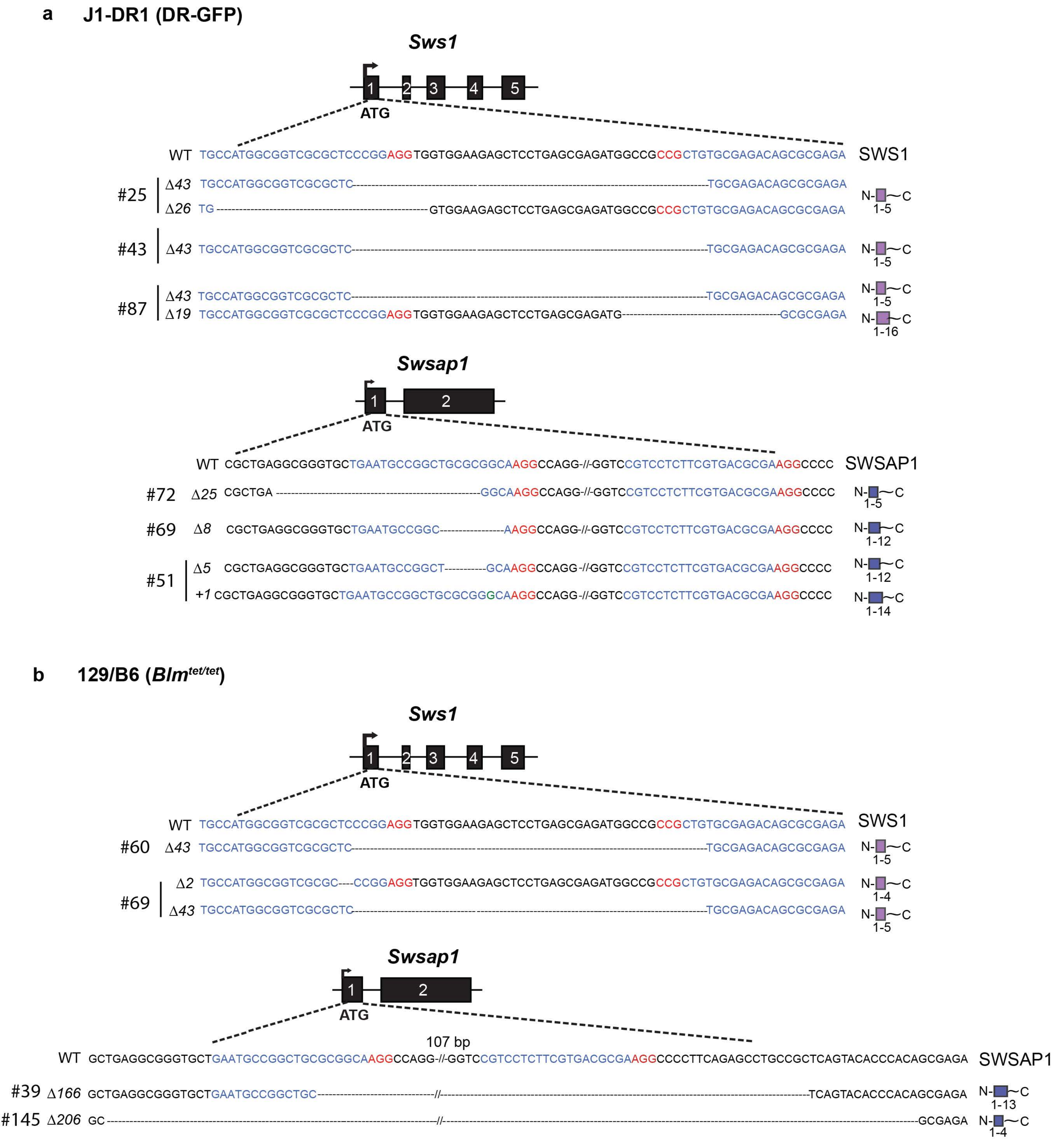

Supplementary Fig. 5: Mutated *Sws1* and *Swsap1* alleles in mouse ES cells.

a,b. *Sws1* and *Swsap1* frameshift mutations in J1-DR1 (a) and 129/B6 (b) ES cell lines that were analyzed, which contain the DR-GFP and IH-HR reporters, respectively. Genomic structure and sequence around the gRNA (blue) and PAM (red) sequences are shown. The number for each mutant clone is shown on the left, together with the detected mutated allele(s). The truncated protein product predicted from each mutated allele is diagrammed on the right.

Supplementary Figure 6

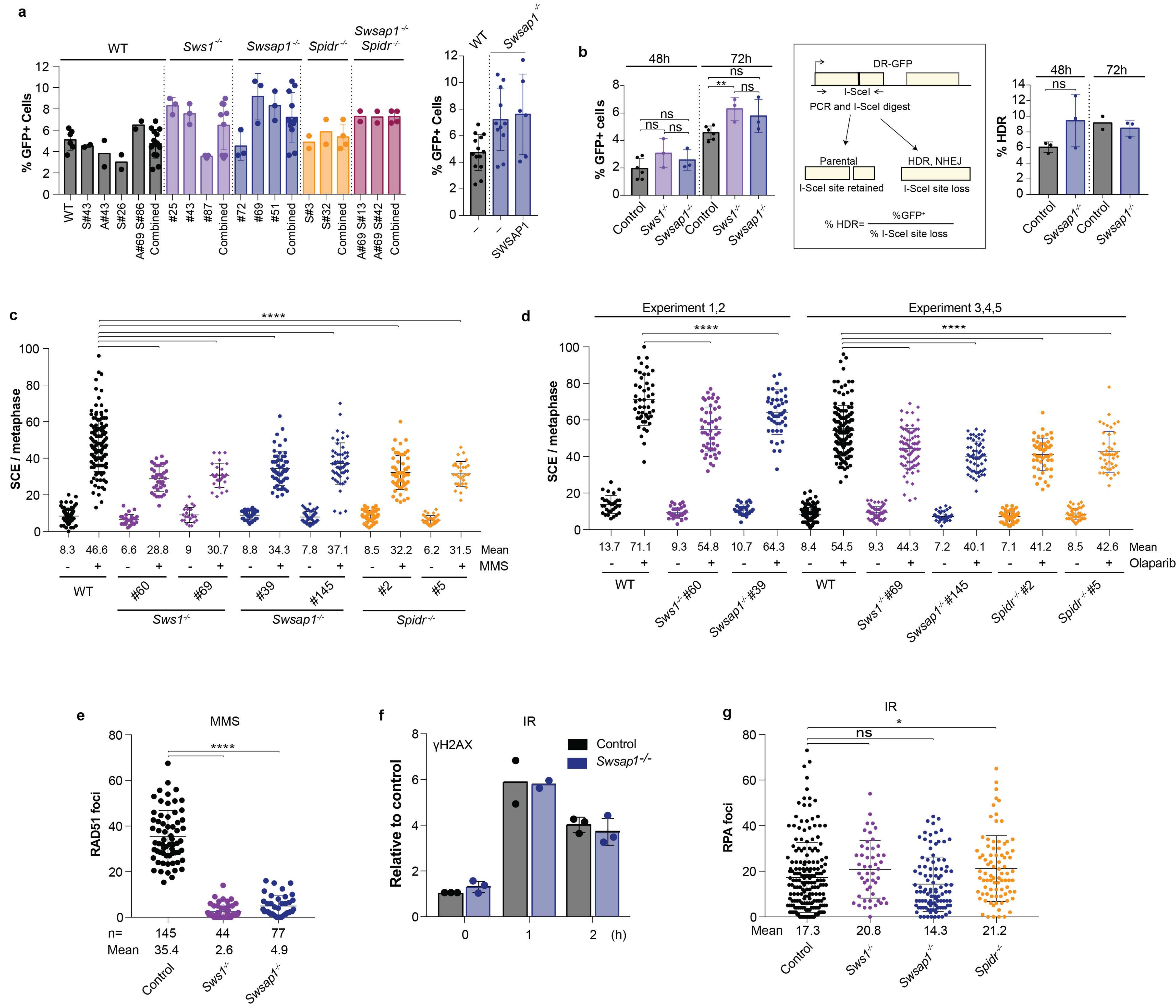

Supplementary Fig. 6: SWS1-SWSAP1-SPIDR promote distinct types of HDR in mitotically-dividing mouse ES cells.

a. Left, HDR results for individual clones using the DR-GFP reporter in wild-type, *Sws1*$^{-/-}$, *Swsap1*$^{-/-}$, *Spidr*$^{-/-}$, and *Swsap1*$^{-/-}$*Spidr*$^{-/-}$ ES cells for data presented in **Fig. 1e**. Wild-type clones includes parental J1 ES cells containing the DR-GFP reporter (J1-DR1) and four subclones isolated after Cas9 expression which maintained wild-type sequences after gRNAs to *Sws1* (S#43), *Swsap1* (A#43), *Spidr* (S#26), and *Swsap1* (A#43) plus *Spidr* (A#69 S#86). Right, Expression of SWSAP1 does not alter HDR in *Swsap1*$^{-/-}$ mutant cells. WT (WT n=6, S#43 n=2, A#43 n=2, S#26 n=2, A#69 S#86 n=2, combined n=14) *Sws1*$^{-/-}$ (#25 n=3, #43 n=3, #87 n=3, combined n=9), *Swsap1*$^{-/-}$ (#72 n=3, #69 n=5, #51 n=3, combined n=11), *Spidr*$^{-/-}$ (#3 n=2, #32 n=2, combined n=4) and *Swsap1*$^{-/-}$*Spidr*$^{-/-}$ (A#69 S#13 n=2, A#69 S#42 n=2, combined n=4), where n is the number of independent experiments.

b. *Sws1*$^{-/-}$and *Swsap1*$^{-/-}$ primary ear fibroblasts have similar level of GFP positive cells as the control at 48 h and 72 h post infection with the I-SceI expression vector. Taking I-SceI-site loss into account to quantify both NHEJ and HDR, the *Swsap1*$^{-/-}$ primary ear fibroblasts have similar HDR levels as control cells. Control (48h, 72h) n=6, *Sws1*$^{-/-}$ (48h, 72h) n=3, *Swsap1*$^{-/-}$ (48h, 72h) n=3, where n is the number of independent experiments.

c,d. SCEs per metaphase for individual clones for data presented in **Fig. 1g** (c) and **Fig. 1h** (d) in untreated conditions and after exposure to MMS (0.5 mM) and olaparib (20 nM). With olaparib exposure, the mean SCEs was higher in the first two experiments and so results are presented separately from subsequent experiments. **c.** WT -MMS n=5, WT +MMS n=6, *Sws1*$^{-/-}$ #60-MMS n=2, *Sws1*$^{-/-}$ #60+MMS n=3, *Sws1*$^{-/-}$ #69-MMS n=2, *Sws1*$^{-/-}$ #69+MMS n=2, *Swsap1*$^{-/-}$ #39-MMS n=2, *Swsap1*$^{-/-}$ #39+MMS n=3, *Swsap1*$^{-/-}$ #145-MMS n=4, *Swsap1*$^{-/-}$ #145+MMS n=5, *Spidr*$^{-/-}$ #2-MMS n=2, *Spidr*$^{-/-}$ #2+MMS n=2, *Spidr*$^{-/-}$ #5-MMS n=1, *Spidr*$^{-/-}$ #5+MMS n=1. **d.** WT-olaparib n=6, WT +olaparib n=7, *Sws1*$^{-/-}$ #60-olaparib n=1, *Sws1*$^{-/-}$ +60+olaparib n=2, *Sws1*$^{-/-}$ #69-olaparib n=2, *Sws1*$^{-/-}$ +69+olaparib n=2, *Swsap1*$^{-/-}$ #39-olaparib n=1, *Swsap1*$^{-/-}$ #39+olaparib n=2, *Swsap1*$^{-/-}$ #145-olaparib n=2, *Swsap1*$^{-/-}$ #145+olaparib n=2, *Spidr*$^{-/-}$ #2-olaparib n=2, *Spidr*$^{-/-}$ #2+olaparib n=2, *Spidr*$^{-/-}$ #5-olaparib n=2, *Spidr*$^{-/-}$ #5+olaparib n=2, where n is the number of independent experiments.

e. *Sws1*$^{-/-}$ and *Swsap1*$^{-/-}$ primary ear fibroblasts have reduced RAD51 focus formation compared to the control cells upon exposure to MMS (0.5 mM). n=3, where n is the number of independent experiments.

f. *Swsap1*$^{-/-}$ and control primary ear fibroblasts have similar levels of DNA damage upon exposure to IR (10 Gy) at various time points. n=3, where n is the number of independent experiments.

g. *Sws1*$^{-/-}$, *Swsap1*$^{-/-}$, *Spidr*$^{-/-}$, and control primary ear fibroblasts have similar number of RPA foci after treatment with IR (10 Gy, 2 h). n=3, where n is the number of independent experiments.

Error bars in **b-f**,g mean ± s.d. *$P \leq 0.05$; **$P \leq 0.01$; ****$P \leq 0.0001$; unpaired t test, two-tailed.

All source data are provided in the Source Data file.

Supplementary Figure 7

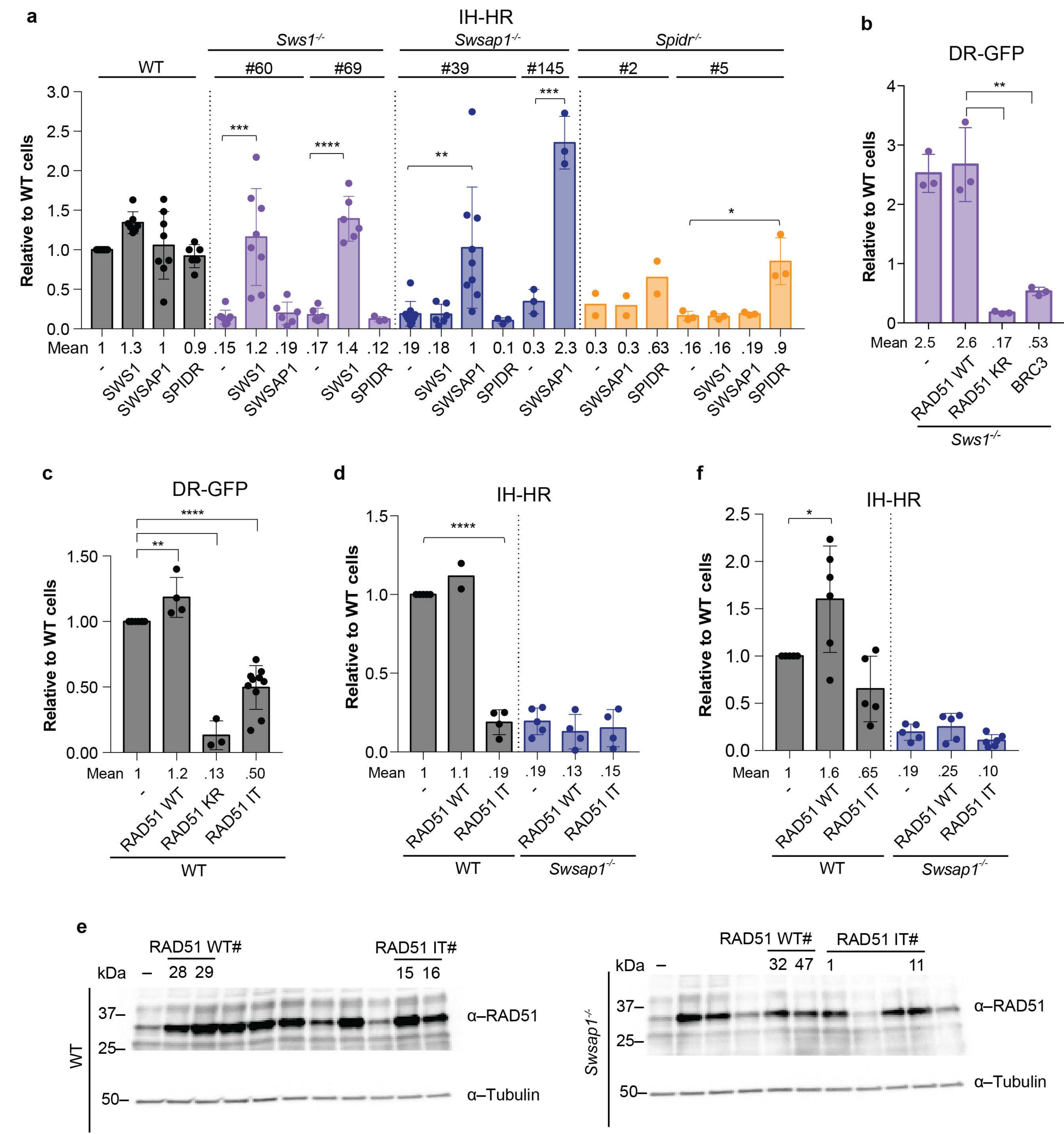

Supplementary Fig. 7: IH-HR analysis in *Sws1$^{-/-}$*, *Swsap1$^{-/-}$*, and *Spidr$^{-/-}$* ES cells.

a. IH-HR in individual clones for data presented in **Fig. 2b**. Relative to WT cells, colony counts expressed relative to wild-type cells transfected with an empty vector within each experiment.

b. In *Sws1$^{-/-}$* cells, RAD51 K133R (KR) and BRC3 peptide expression reduces HDR in DR-GFP to a similar extent as in wild-type and *Swsap1$^{-/-}$* cells. (Compare with Fig. 2d).

c. Transient overexpression of RAD51 I287T (IT) reduces HDR in wild-type cells using the DR-GFP reporter, although to a lesser extent than RAD51 K133R.

d. Transient overexpression of RAD51 I287T (IT) does not restore IH-HR in *Swsap1$^{-/-}$* cells, and reduces IH-HR in wild-type cells. n=3, where n is the number of independent experiments.

e. Western blots showing RAD51 levels in the untargeted (-) and targeted clones at the *Rosa26* locus for each RAD51 WT and RAD51 I287T (IT) in wild-type and *Swsap1$^{-/-}$* cells. Two clones were chosen for each which had approximately doubled the total amount of endogenous RAD51, as indicated.

f. Constitutive overexpression of RAD51 WT increases IH-HR in wild-type and *Swsap1$^{-/-}$* cells, while RAD51 I287T expression reduces IH-HR. . WT n=5, WT+RAD51WT n=6, WT+RAD51IT n=5, *Swsap1$^{-/-}$* n=5, *Swsap1$^{-/-}$* +RAD51WT n=5, *Swsap1$^{-/-}$* +RAD51IT n=6, where n is the number of independent experiments. This graph uses the same data as in **Fig. 2f** but here the data are presented relative to wild-type cells that are transfected with an empty vector (-).

Error bars in **a-d**,f mean ± s.d. *$P \leq 0.05$; **$P \leq 0.01$; ***$P \leq 0.001$; ****$P \leq 0.0001$; unpaired t test, two-tailed.

All source data are provided in the Source Data file.

Supplementary Figure 8

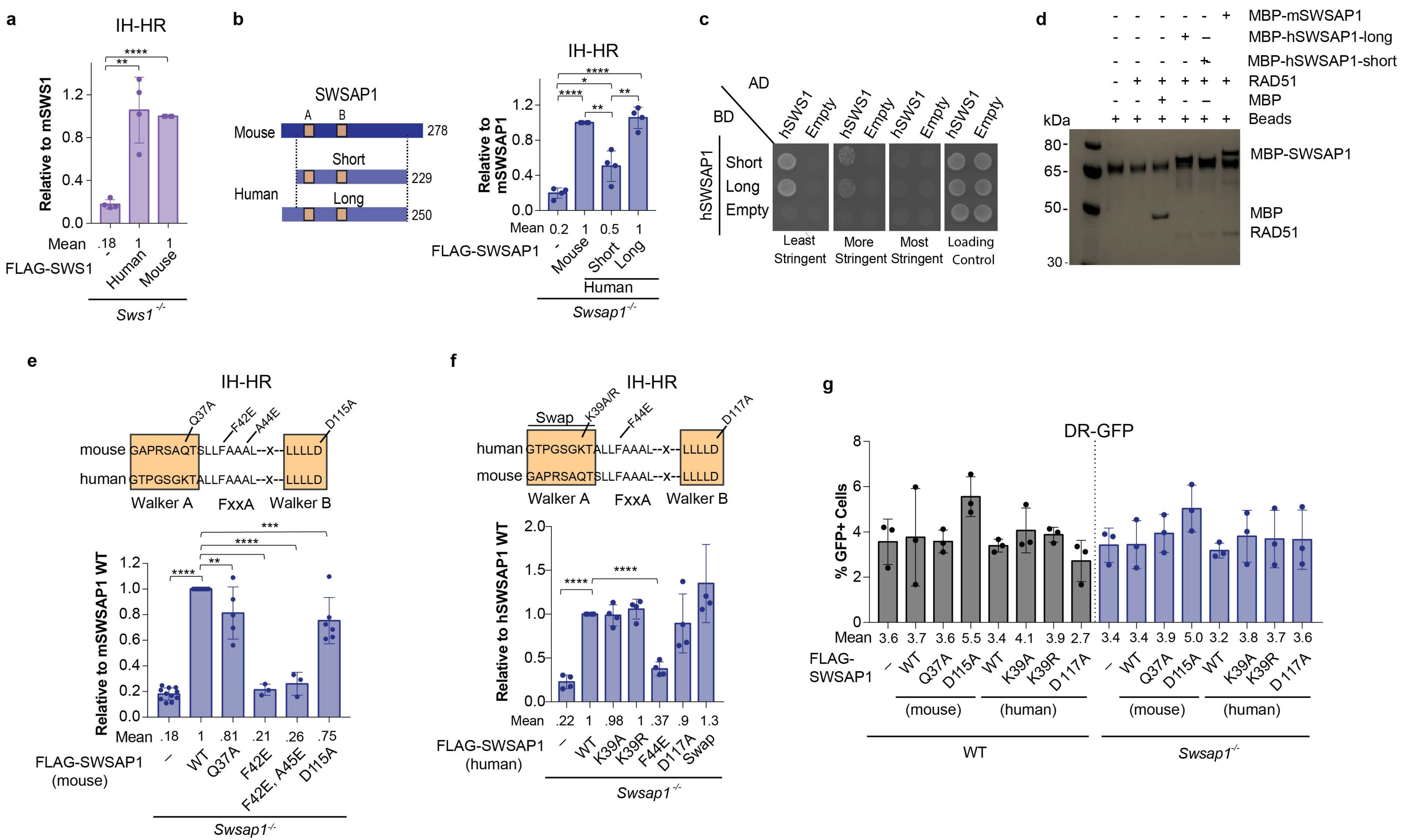

Supplementary Fig. 8: SWSAP1 requirements to promote IH-HR.

a. Both human and mouse SWS1 complement the IH-HR defects of mouse *Sws1*$^{-/-}$ cells to a similar level. n=4, where n is the number of independent experiments.

b. The human long form of SWSAP1 complements the IH-HR defect of mouse *Swsap1*$^{-/-}$ cells to a similar extent as mouse SWSAP1, while the short form has reduced activity. n=4, where n is the number of independent experiments.

c. Yeast-two-hybrid analysis of the short and long forms of human SWSAP1 interaction with human SWS1. Interaction was assessed with SWS1 fused to the GAL4 activation domain (AD) and SWSAP1 short or long form fused to the GAL4-DNA binding domain (BD). Strength of the interaction was assessed by plating on different plates [least stringent (SC-HIS-LEU-TRP), more stringent (SC-HIS-LEU-TRP+3AT), and most stringent (SC-HIS-LEU-TRP-ADE)]. Cells were plated on SC-LEU-TRP as a loading control and the empty vectors were used as negative controls.

d. Recombinant long and short isoforms of human SWSAP1 as well as recombinant mouse SWSAP1 bind to RAD51 *in vitro*. n=3, where n is the number of independent experiments.

e,f. Analysis of mouse (e) and human (f) SWSAP1 mutants for IH-HR activity in mouse *Swsap1*$^{-/-}$ cells. Sequence alignment between mouse and human SWSAP1 shows the conservation in Walker A, Walker B, and RAD51 binding motifs and the residues mutated in this study. Mutations in the Walker motifs (e,f) or "Swap" of the human Walker A motif with the mouse sequence (4 amino acid substitutions to the human protein) (f) have little or no effect on IH-HR. By contrast, mutation of the phenylalanine in the RAD51 binding motif greatly reduces IH-HR, although additional mutation of the alanine in the FxxA motif has no further effect. e; *Swsap1*$^{-/-}$, *Swsap1*$^{-/-}$ +SWSAP1-WT n=11, *Swsap1*$^{-/-}$ +SWSAP1-Q37A n=5, *Swsap1*$^{-/-}$ +SWSAP1-F42E, *Swsap1*$^{-/-}$ +SWSAP1-F42E, A45E n=3, *Swsap1*$^{-/-}$ +SWSAP1-D115A n=6, f; n=4, where n is the number of independent experiments.

g. Analysis of mouse and human SWSAP1 Walker A and B mutants in the DR-GFP assay in wild-type and *Swsap1*$^{-/-}$ cells, showing no significant effects. n=3, where n is the number of independent experiments.

Error bars in **a,b,e-g**, mean ± s.d. *$P \leq 0.05$; **$P \leq 0.01$; ***$P \leq 0.001$; ****$P \leq 0.0001$; unpaired t test, two-tailed.

All source data are provided in the Source Data file.

Supplementary Figure 9

a

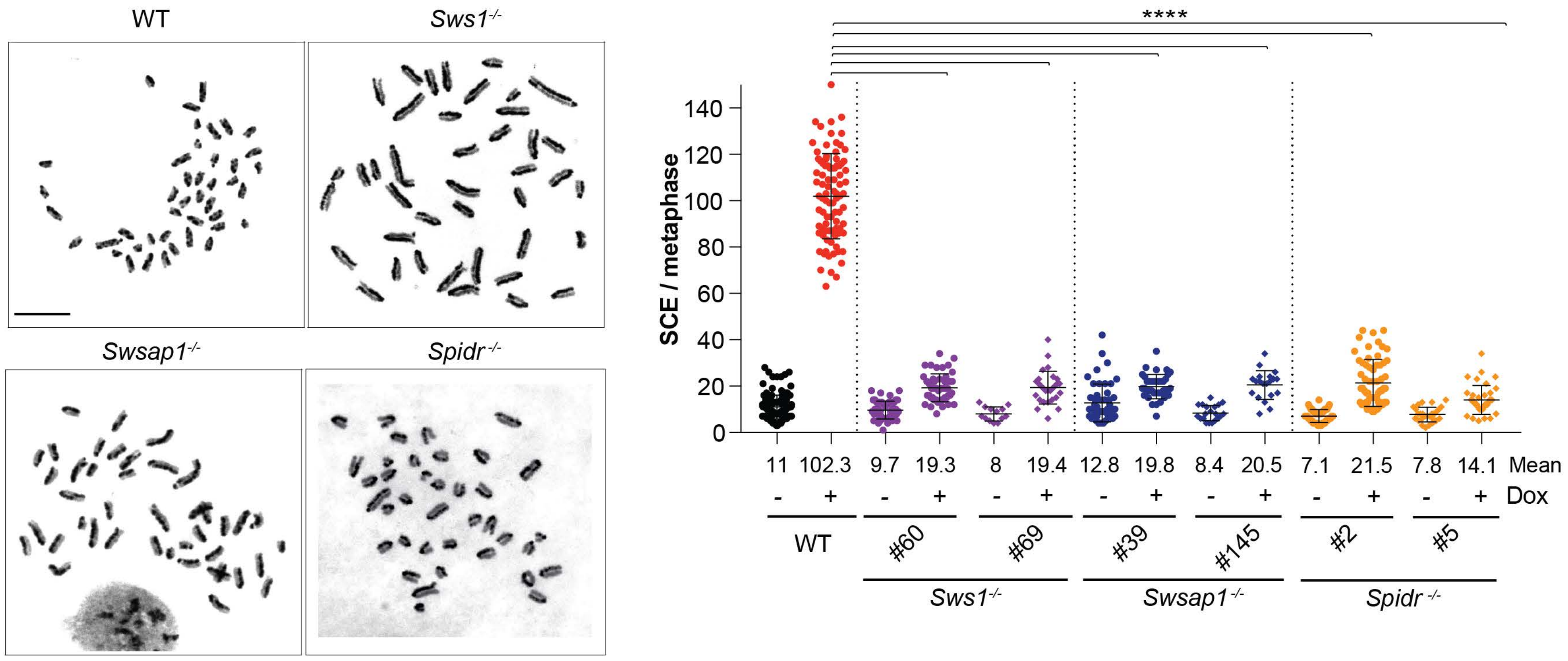

b

BLM	Cell line	Total *neo*$^{+}$ clones	Clones w/o LOH	Clones with LOH	P value (vs control)	P value (-/+ Dox)
-Dox	Control	288	268	20	–	–
	Sws1$^{-/-}$	288	282	6	0.0079	–
	Swsap1$^{-/-}$	288	283	5	0.0033	–
+Dox	Control	297	247	50	–	0.0003
	Sws1$^{-/-}$	336	321	15	0.0001	0.1208
	Swsap1$^{-/-}$	336	317	19	0.0001	0.0118

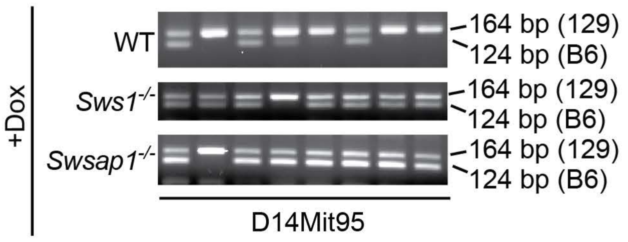

c

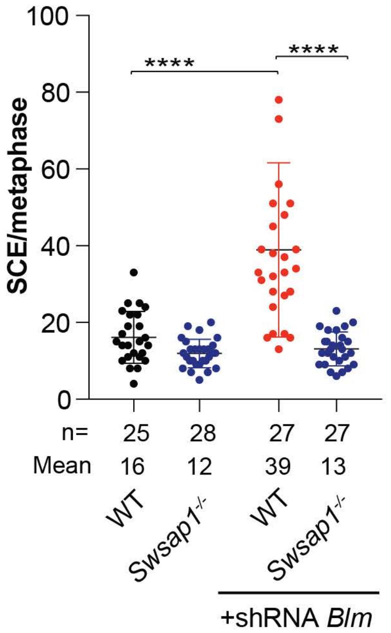

d

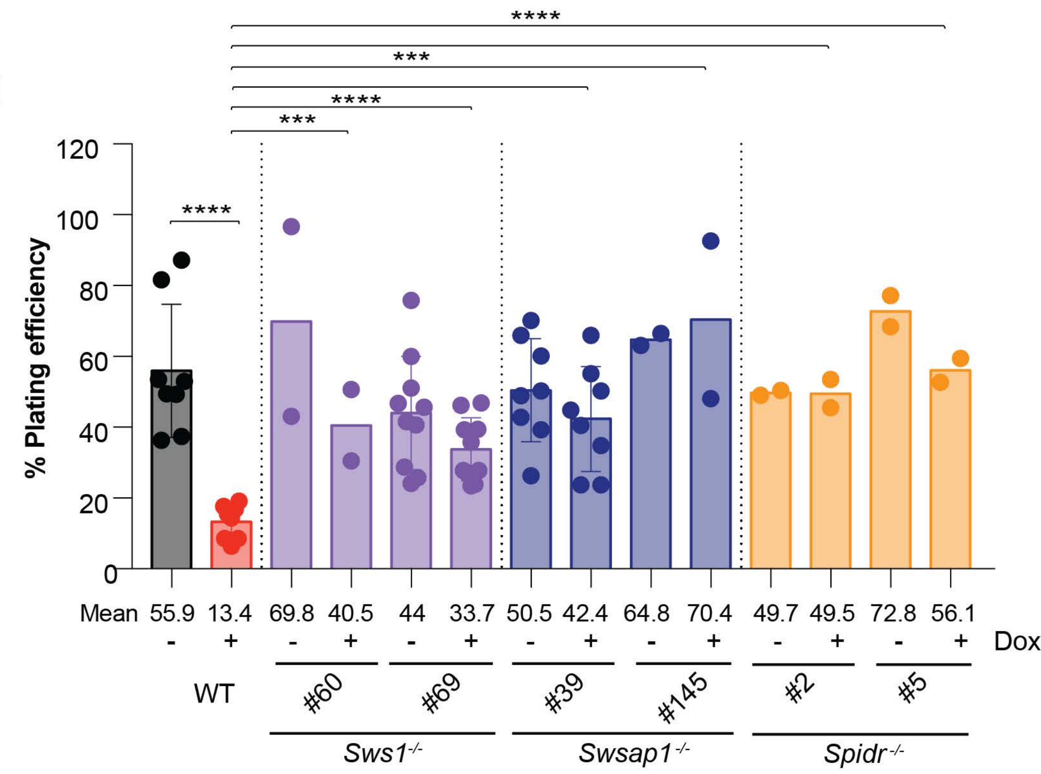

e

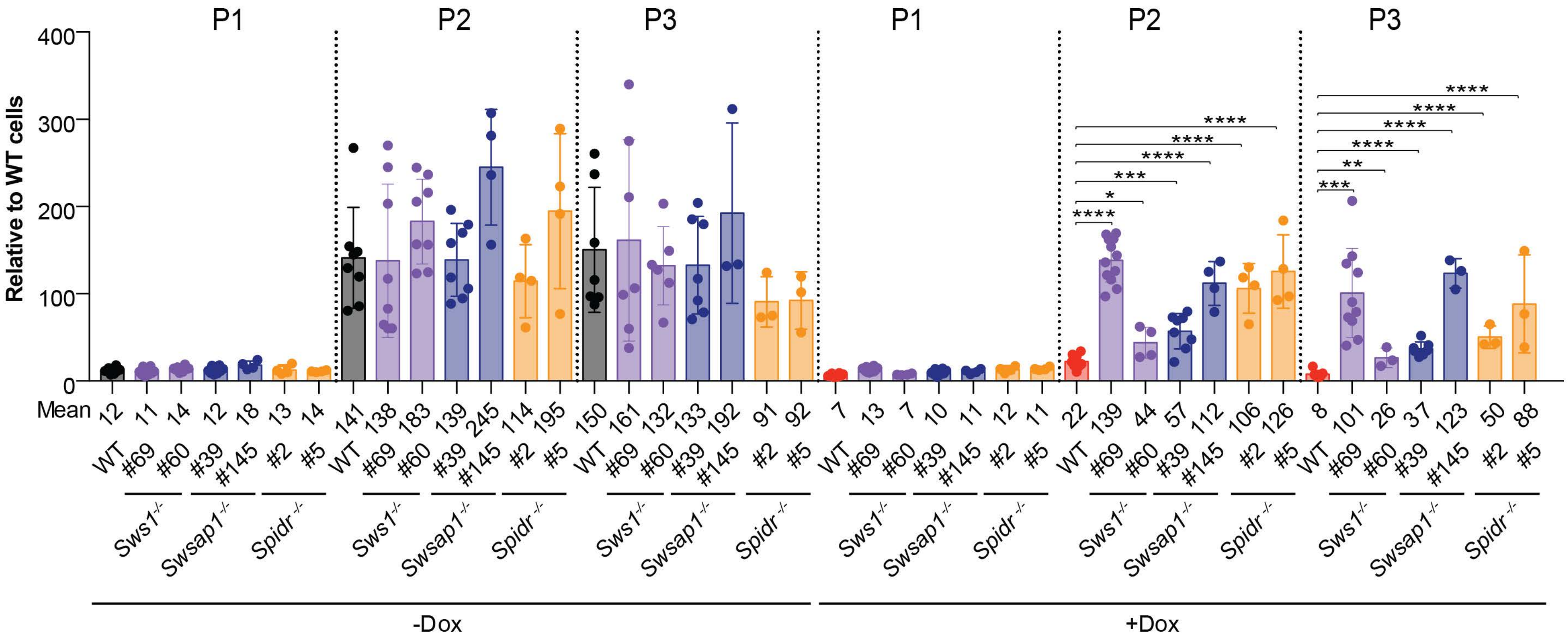

f

Weaning (21d)	Control				*Swsap1*$^{-/-}$		*Blm*$^{-/-}$		*Swsap1*$^{-/-}$ *Blm*$^{-/-}$
**Total (122)**	**wt/wt; +/+**	**wt/wt; +/-**	**Δ/wt; +/+**	**Δ/wt; +/-**	**Δ/Δ; +/+**	**Δ/Δ ; +/-**	**wt/wt ; -/-**	**Δ/wt; -/-**	**Δ/Δ ; -/-**
Observed	7.3% (9)	17.2% (21)	14% (17)	37.7% (46)	4.1% (5)	19.7% (24)	0% (0)	0% (0)	0% (0)
Expected	6.25% (7)	12.50% (15)	12.50% (15)	25% (30)	6.25% (7)	12.50% (15)	6.25% (7)	12.50% (15)	6.25% (7)

g

Embryo	Control				*Swsap1*$^{-/-}$		*Blm*$^{-/-}$		*Swsap1*$^{-/-}$ *Blm*$^{-/-}$	
**E15.5 (196)**	**wt/wt; +/+**	**wt/wt; +/-**	**Δ/wt; +/+**	**Δ/wt; +/-**	**Δ/Δ; +/+**	**Δ/Δ ; +/-**	**wt/wt ; -/-**	**Δ/wt; -/-**	**Δ/Δ ; -/-**	**Resorbed**
Observed	9.2% (18)	18.8% (37)	9.2% (18)	31.1% (61)	6.1% (12)	16.3% (32)	1.5% (3)	2.5% (5)	5.1% (10)	18
Expected	6.25% (12)	12.50% (24)	12.50% (24)	25% (48)	6.25% (12)	12.50% (24)	6.25% (12)	12.50% (24)	6.25% (12)	

Supplementary Fig. 9: Loss of SWS1-SWSAP1-SPIDR reduces SCEs and LOH, and enhances cell proliferation in the absence of BLM.

a. SCE analysis in *Blm*$^{tet/tet}$ cells after Dox exposure to deplete BLM. Representative metaphase chromosome spreads are shown along with quantification from individual clones for data presented in **Fig. 3a**. WT-dox n=7, WT +dox n=7, *Sws1*$^{-/-}$ #60-dox n=3, *Sws1*$^{-/-}$ #60+dox n=3, *Sws1*$^{-/-}$ #69-dox n=2, *Sws1*$^{-/-}$ #69+dox n=2, *Swsap1*$^{-/-}$ #39-dox n=3, *Swsap1*$^{-/-}$ #39+dox n=3,*Swsap1*$^{-/-}$ #145-dox n=1, *Swsap1*$^{-/-}$ #145+dox n=1, *Spidr*$^{-/-}$ #2-dox n=3, *Spidr*$^{-/-}$ #2+dox n=3, *Spidr*$^{-/-}$ #5-dox n=2, *Spidr*$^{-/-}$ #5+dox n=2, where n is the number of independent experiments.

b. LOH of the distal marker D14Mit95 is reduced in cells mutated for *Sws1* or *Swsap1*. Parental cells are heterozygous for the 129 and B6 alleles of this marker, but *neo*$^{+}$ colonies that have undergone IH-HR with LOH have a single 129 or B6 allele. Total *neo*+ clones combined from four experiments which showed some variability. See Supplementary Data File. n=4, where n is the number of independent experiments. Fisher's exact test.

c. Knockdown of BLM in wild-type MEFs leads to an increase in SCE formation, however, *Swsap1*$^{-/-}$ MEFs depleted for BLM show reduced SCE. n=2, where n is the number of independent experiments.

d. Plating efficiency for wild-type, *Sws1*$^{-/-}$, *Swsap1*$^{-/-}$, and *Spidr*$^{-/-}$ cells with and without Dox for data presented in **Fig. 3f**. n=8, where n is the number of independent experiments.

e. Population doubling for individual clones for data presented in **Fig. 3g**. n=6, where n is the number of independent experiments.

f. Breeding analysis showing that *Blm*$^{-/-}$ and *Swsap1*$^{-/-}$ *Blm*$^{-/-}$ mice are not viable upon weaning (21d). Genotypes for *Swsap1* are shown first followed by genotypes for *Blm*.

g. Timed matings showing that *Swsap1*$^{-/-}$ *Blm*$^{-/-}$ embryos are obtained at nearly normal Mendelian ratios at E15.5, whereas *Blm*$^{-/-}$ single mutants are highly underrepresented. Partial data from this table are presented in **Fig. 4a**.

Error bars in a,**c-e**, mean ± s.d. *$P \leq 0.05$; **$P \leq 0.01$; ***$P \leq 0.001$; ****$P \leq 0.0001$; unpaired t test, two-tailed.

All source data are provided in the Source Data file.

Supplementary Figure 10

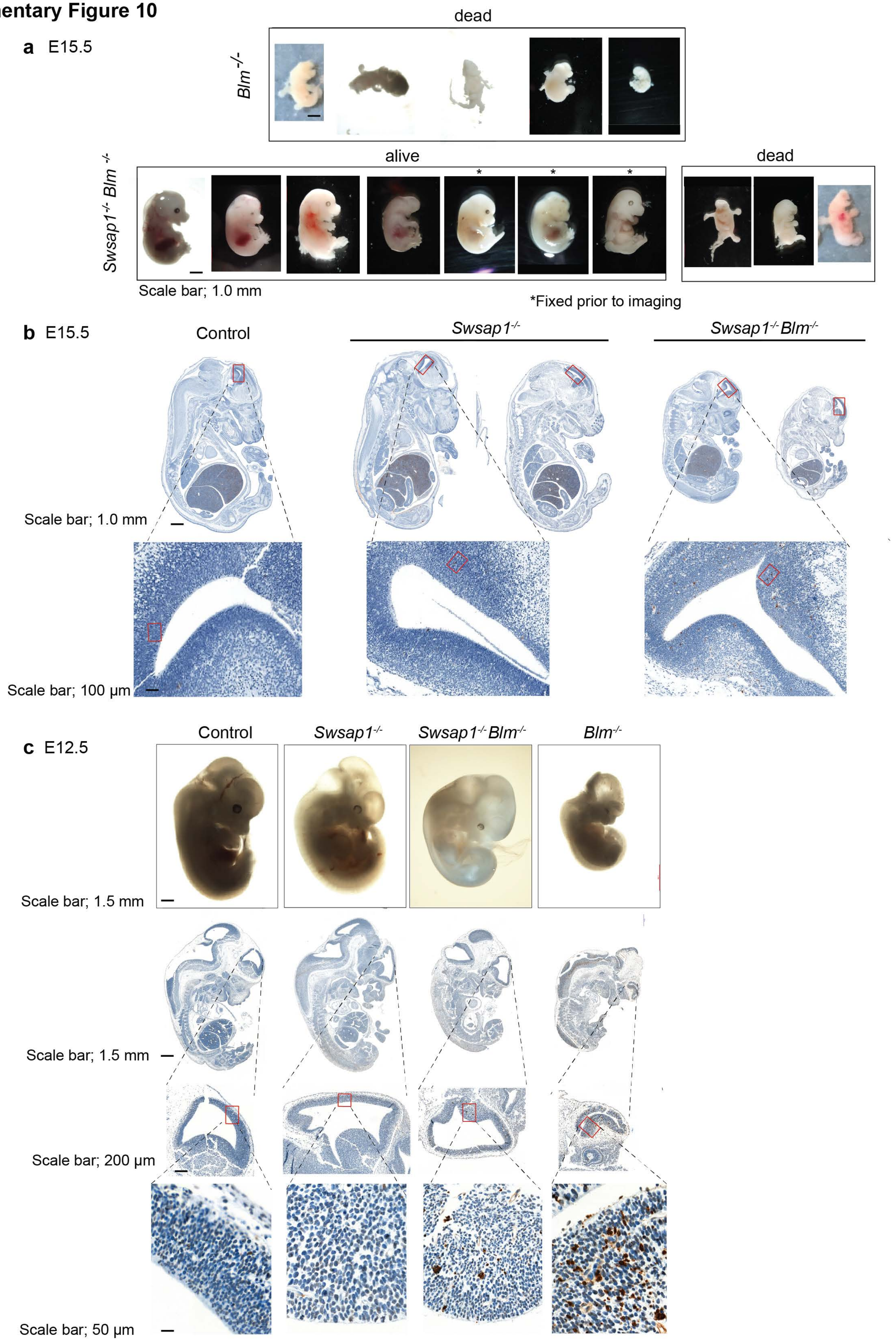

Supplementary Fig. 10: Loss of SWSAP1 prolongs survival of *Blm* mutant embryos.

a. Images of all E15.5 *Swsap1*$^{-/-}$ *Blm*$^{-/-}$ and *Blm*$^{-/-}$ embryos for data presented in **Fig. 4b**. Scale bar, 1 mm. *Swsap1*$^{-/-}$ *Blm*$^{-/-}$ embryos fixed in PFA for TUNEL analysis before the images were taken are indicated.

b. TUNEL-stained sections of E15.5 embryos. Red rectangles in bottom forebrain images show regions in embryo sections that were displayed in lower panels of **Fig. 4b**.

c. Images of E12.5 embryos and TUNEL-stained sections. Scale bar, 1.5 mm. The *Swsap1*$^{-/-}$ *Blm*$^{-/-}$ embryo appears better developed compared to the *Blm*$^{-/-}$ embryo and has fewer TUNEL-stained cells, although more than the *Swsap1*$^{-/-}$ and control embryos.
